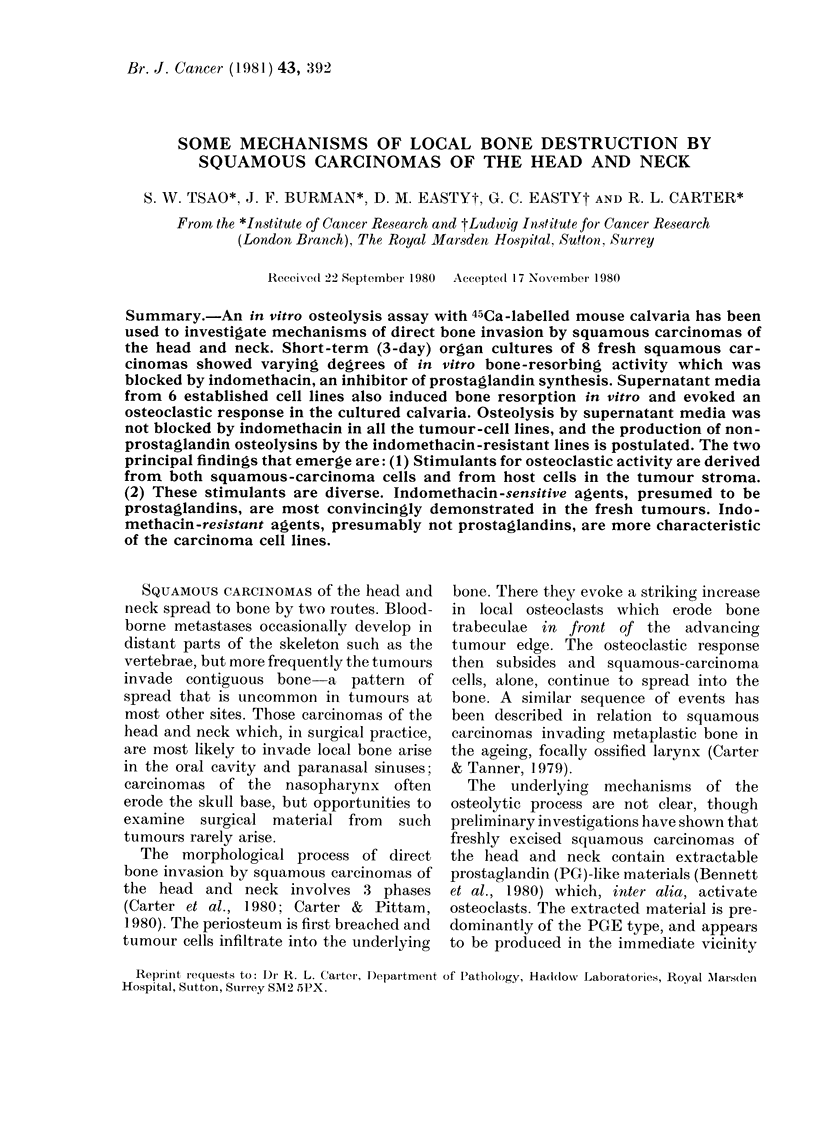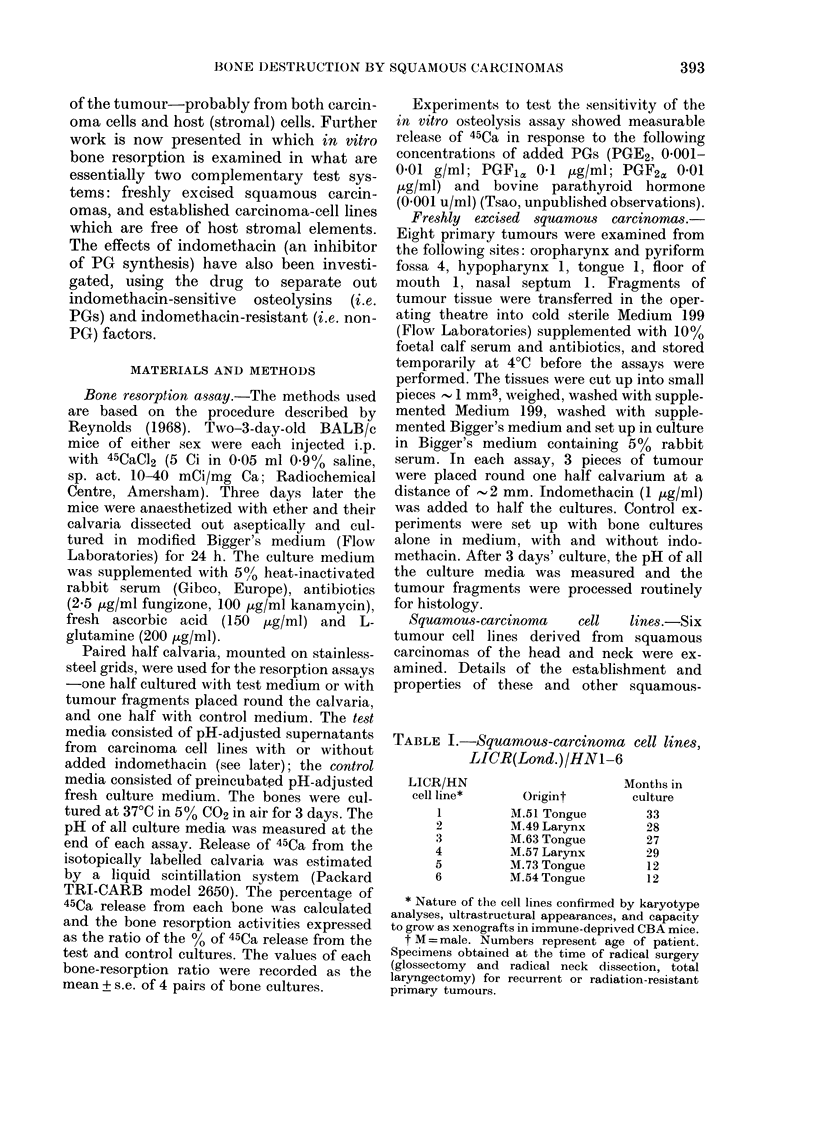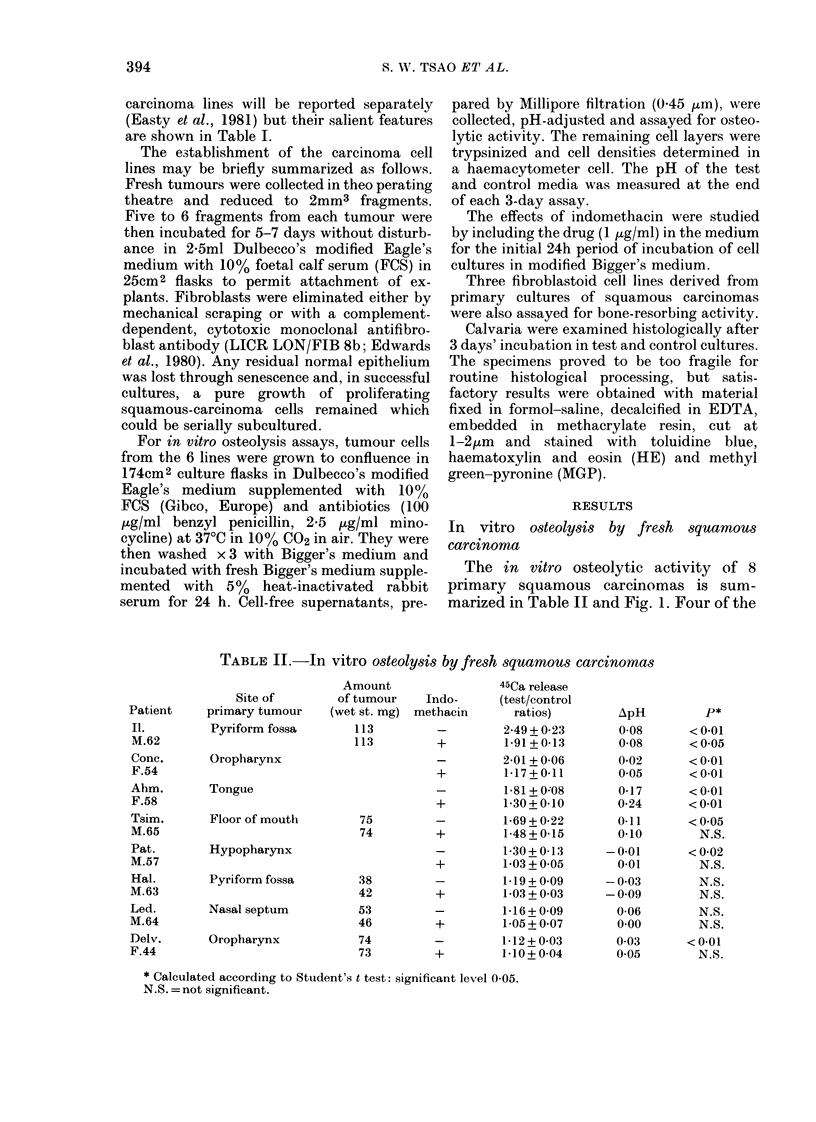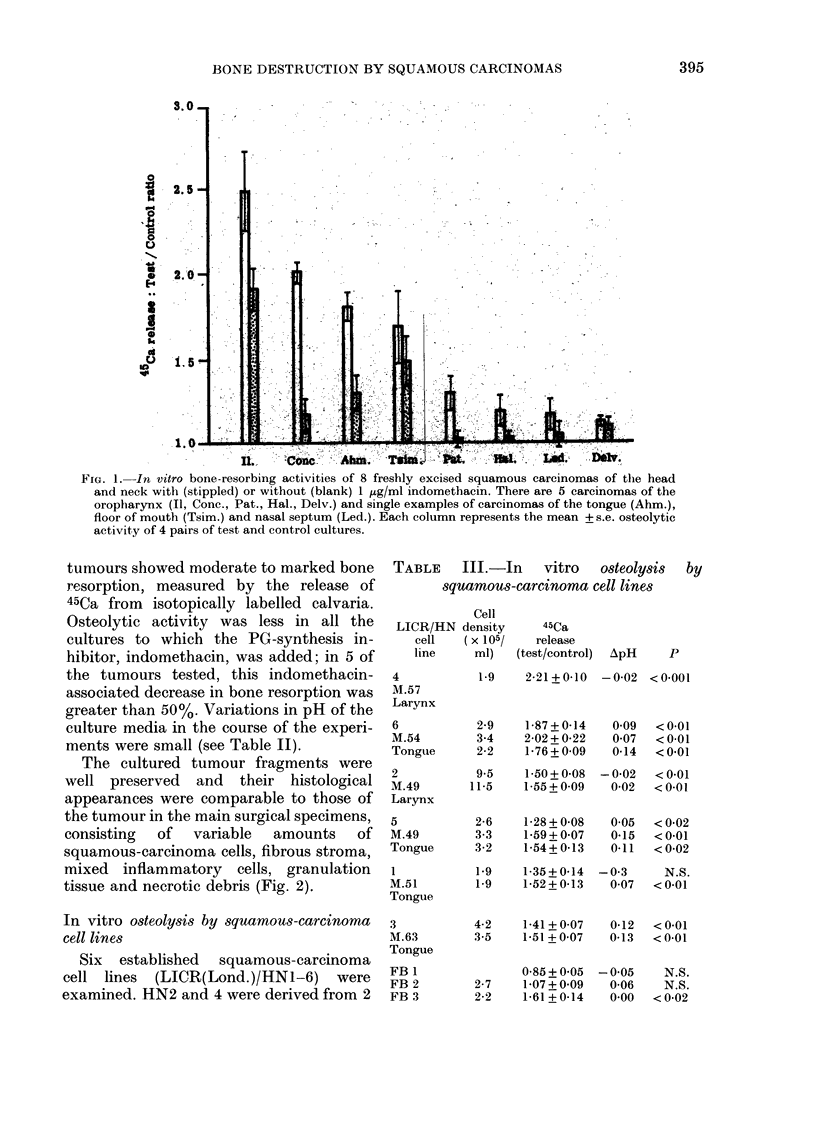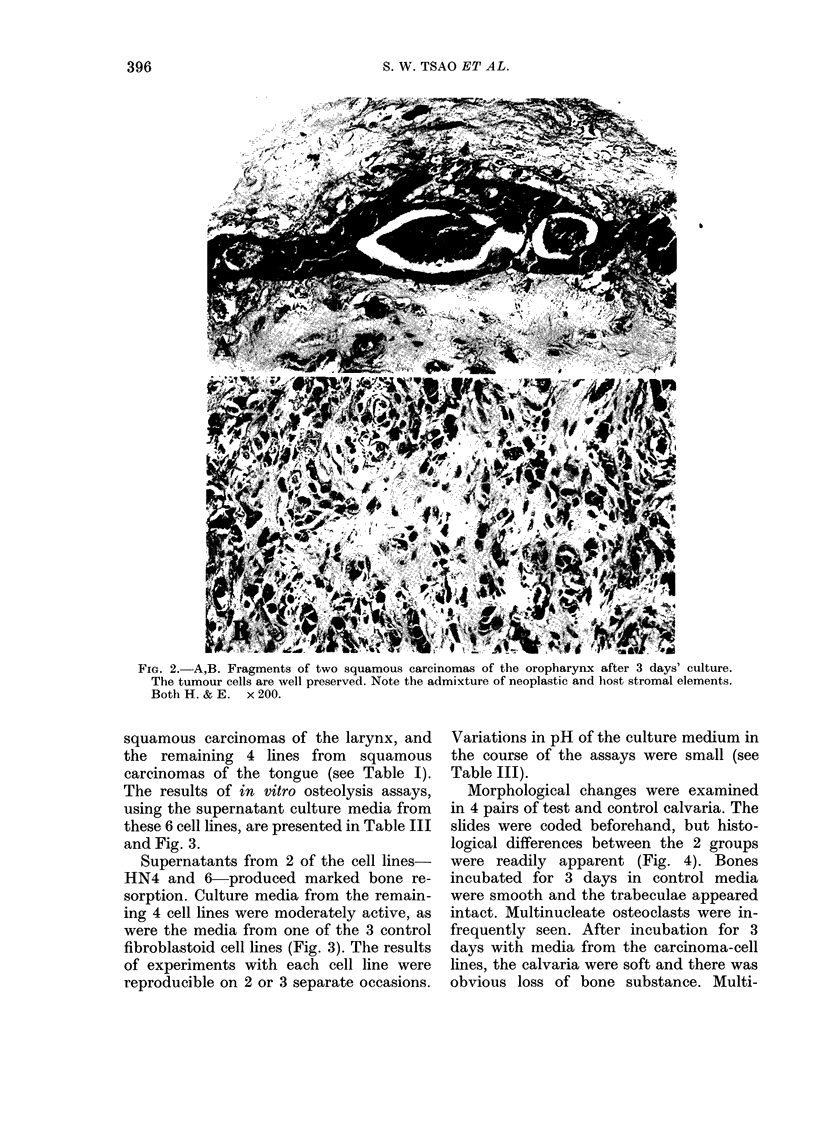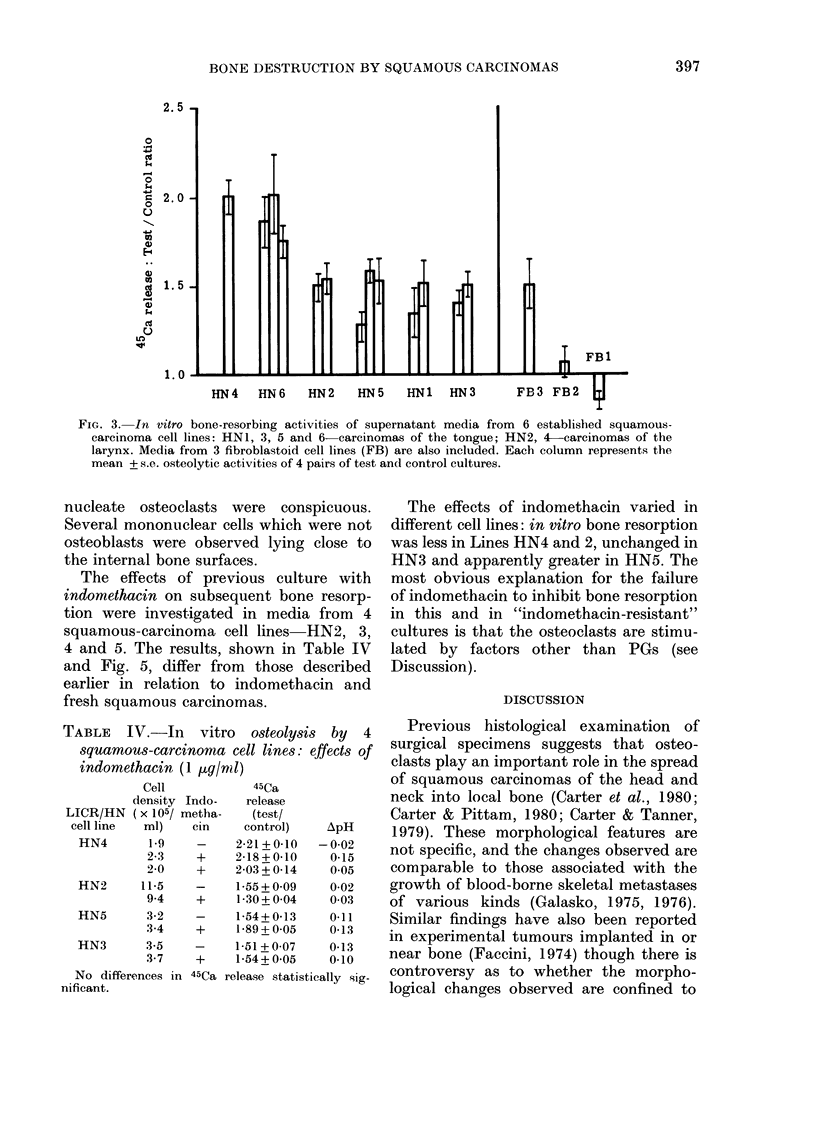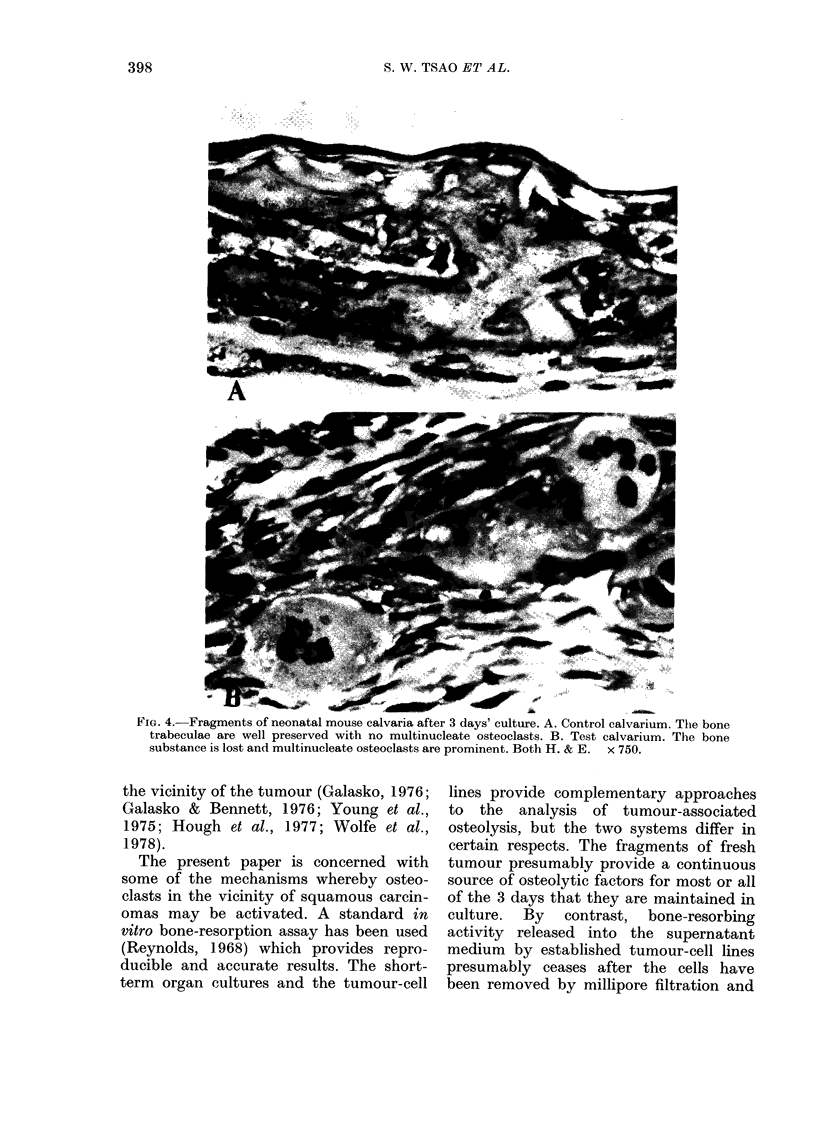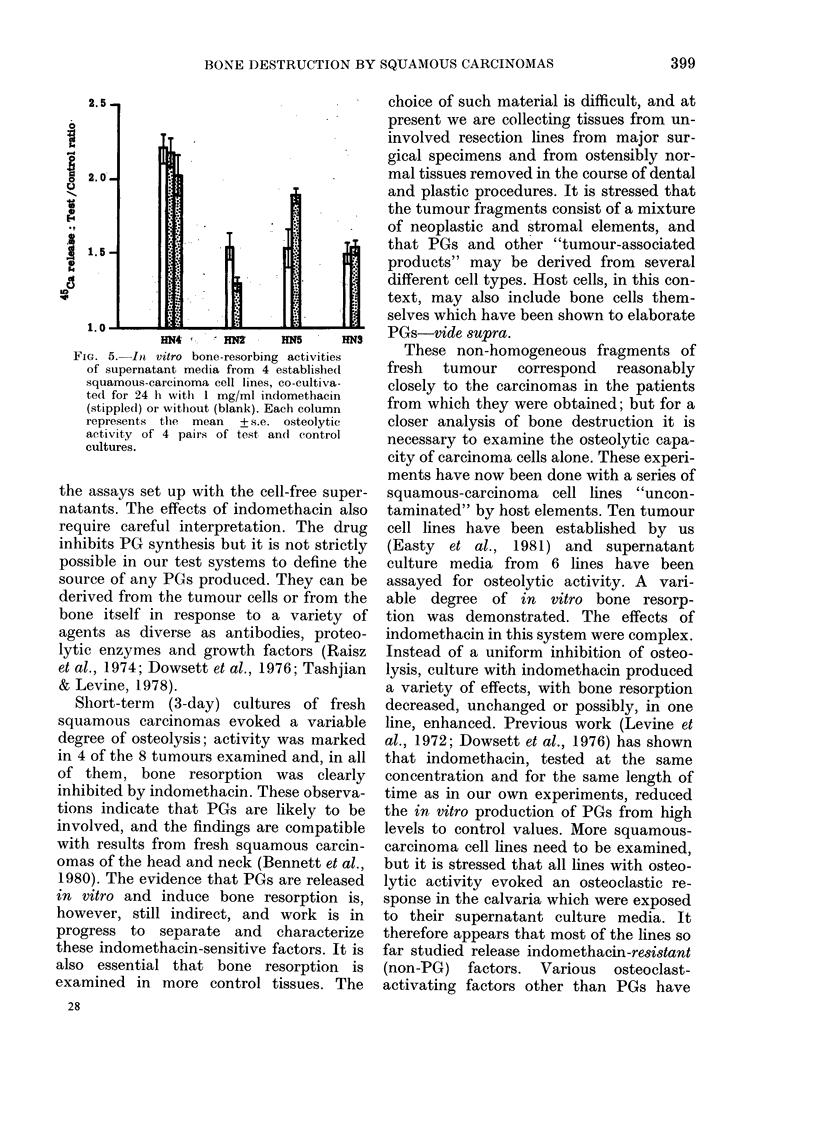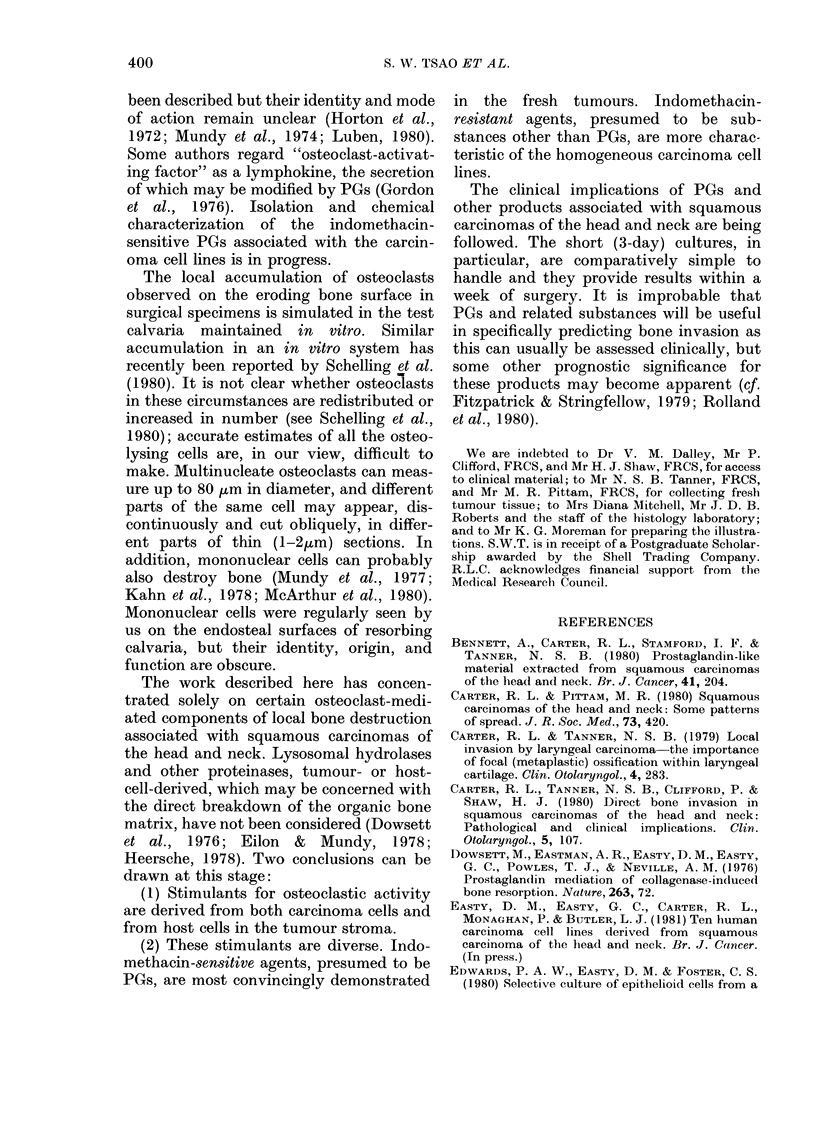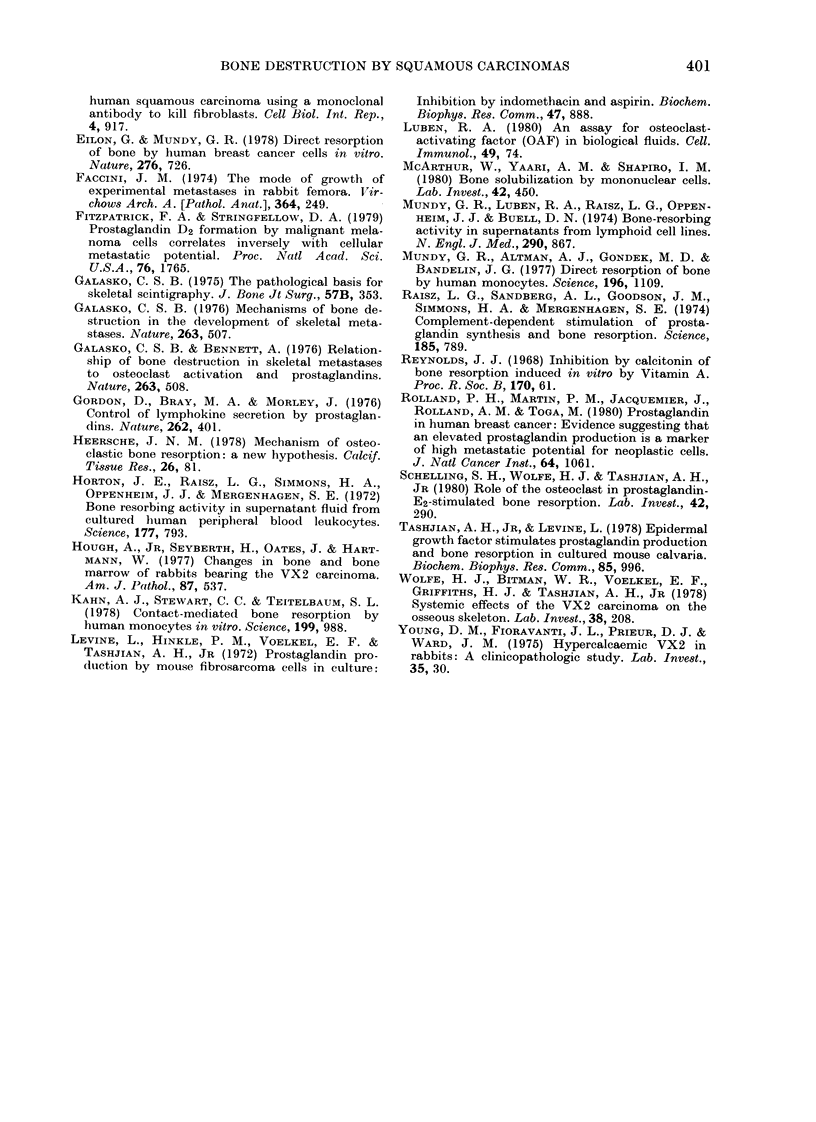# Some mechanisms of local bone destruction by squamous carcinomas of the head and neck.

**DOI:** 10.1038/bjc.1981.60

**Published:** 1981-03

**Authors:** S. W. Tsao, J. F. Burman, D. M. Easty, G. C. Easty, R. L. Carter

## Abstract

**Images:**


*Br. J. Cancer* (1981) **43**, 392

### SOME MECHANISMS OF LOCAL BONE DESTRUCTION BY SQUAMOUS CARCINOMAS OF THE HEAD AND NECK

S. W. TSAO*, J. F. BURMAN*, D. M. EASTY†, G. C. EASTY† AND R. L. CARTER*

*From the *Institute of Cancer Research and †Ludwig Institute for Cancer Research (London Branch), The Royal Marsden Hospital, Sutton, Surrey*

Received 22 September 1980 Accepted 17 November 1980

**Summary.—An *in vitro* osteolysis assay with $^{45}Ca$-labelled mouse calvaria has been used to investigate mechanisms of direct bone invasion by squamous carcinomas of the head and neck. Short-term (3-day) organ cultures of 8 fresh squamous carcinomas showed varying degrees of *in vitro* bone-resorbing activity which was blocked by indomethacin, an inhibitor of prostaglandin synthesis. Supernatant media from 6 established cell lines also induced bone resorption *in vitro* and evoked an osteoclastic response in the cultured calvaria. Osteolysis by supernatant media was not blocked by indomethacin in all the tumour-cell lines, and the production of non-prostaglandin osteolysins by the indomethacin-resistant lines is postulated. The two principal findings that emerge are: (1) Stimulants for osteoclastic activity are derived from both squamous-carcinoma cells and from host cells in the tumour stroma. (2) These stimulants are diverse. Indomethacin-*sensitive* agents, presumed to be prostaglandins, are most convincingly demonstrated in the fresh tumours. Indomethacin-*resistant* agents, presumably not prostaglandins, are more characteristic of the carcinoma cell lines.**

SQUAMOUS CARCINOMAS of the head and neck spread to bone by two routes. Blood-borne metastases occasionally develop in distant parts of the skeleton such as the vertebrae, but more frequently the tumours invade contiguous bone—a pattern of spread that is uncommon in tumours at most other sites. Those carcinomas of the head and neck which, in surgical practice, are most likely to invade local bone arise in the oral cavity and paranasal sinuses; carcinomas of the nasopharynx often erode the skull base, but opportunities to examine surgical material from such tumours rarely arise.

The morphological process of direct bone invasion by squamous carcinomas of the head and neck involves 3 phases (Carter *et al.*, 1980; Carter & Pittam, 1980). The periosteum is first breached and tumour cells infiltrate into the underlying bone. There they evoke a striking increase in local osteoclasts which erode bone trabeculae *in front of* the advancing tumour edge. The osteoclastic response then subsides and squamous-carcinoma cells, alone, continue to spread into the bone. A similar sequence of events has been described in relation to squamous carcinomas invading metaplastic bone in the ageing, focally ossified larynx (Carter & Tanner, 1979).

The underlying mechanisms of the osteolytic process are not clear, though preliminary investigations have shown that freshly excised squamous carcinomas of the head and neck contain extractable prostaglandin (PG)-like materials (Bennett *et al.*, 1980) which, *inter alia*, activate osteoclasts. The extracted material is predominantly of the PGE type, and appears to be produced in the immediate vicinity

Reprint requests to: Dr R. L. Carter, Department of Pathology, Haddow Laboratories, Royal Marsden Hospital, Sutton, Surrey SM2 5PX.

of the tumour—probably from both carcinoma cells and host (stromal) cells. Further work is now presented in which *in vitro* bone resorption is examined in what are essentially two complementary test systems: freshly excised squamous carcinomas, and established carcinoma-cell lines which are free of host stromal elements. The effects of indomethacin (an inhibitor of PG synthesis) have also been investigated, using the drug to separate out indomethacin-sensitive osteolysins (*i.e.* PGs) and indomethacin-resistant (*i.e.* non-PG) factors.

### MATERIALS AND METHODS

*Bone resorption assay.*—The methods used are based on the procedure described by Reynolds (1968). Two–3-day-old BALB/c mice of either sex were each injected i.p. with $^{45}CaCl_2$ (5 Ci in 0·05 ml 0·9% saline, sp. act. 10–40 mCi/mg Ca; Radiochemical Centre, Amersham). Three days later the mice were anaesthetized with ether and their calvaria dissected out aseptically and cultured in modified Bigger's medium (Flow Laboratories) for 24 h. The culture medium was supplemented with 5% heat-inactivated rabbit serum (Gibco, Europe), antibiotics (2·5 $\mu$g/ml fungizone, 100 $\mu$g/ml kanamycin), fresh ascorbic acid (150 $\mu$g/ml) and L-glutamine (200 $\mu$g/ml).

Paired half calvaria, mounted on stainless-steel grids, were used for the resorption assays —one half cultured with test medium or with tumour fragments placed round the calvaria, and one half with control medium. The *test* media consisted of pH-adjusted supernatants from carcinoma cell lines with or without added indomethacin (see later); the *control* media consisted of preincubated pH-adjusted fresh culture medium. The bones were cultured at 37°C in 5% $CO_2$ in air for 3 days. The pH of all culture media was measured at the end of each assay. Release of $^{45}$Ca from the isotopically labelled calvaria was estimated by a liquid scintillation system (Packard TRI-CARB model 2650). The percentage of $^{45}$Ca release from each bone was calculated and the bone resorption activities expressed as the ratio of the % of $^{45}$Ca release from the test and control cultures. The values of each bone-resorption ratio were recorded as the mean $\pm$ s.e. of 4 pairs of bone cultures.

Experiments to test the sensitivity of the *in vitro* osteolysis assay showed measurable release of $^{45}$Ca in response to the following concentrations of added PGs ($PGE_2$, 0·001–0·01 g/ml; $PGF_{1\alpha}$ 0·1 $\mu$g/ml; $PGF_{2\alpha}$ 0·01 $\mu$g/ml) and bovine parathyroid hormone (0·001 u/ml) (Tsao, unpublished observations).

*Freshly excised squamous carcinomas.*—Eight primary tumours were examined from the following sites: oropharynx and pyriform fossa 4, hypopharynx 1, tongue 1, floor of mouth 1, nasal septum 1. Fragments of tumour tissue were transferred in the operating theatre into cold sterile Medium 199 (Flow Laboratories) supplemented with 10% foetal calf serum and antibiotics, and stored temporarily at 4°C before the assays were performed. The tissues were cut up into small pieces ~1 mm³, weighed, washed with supplemented Medium 199, washed with supplemented Bigger's medium and set up in culture in Bigger's medium containing 5% rabbit serum. In each assay, 3 pieces of tumour were placed round one half calvarium at a distance of ~2 mm. Indomethacin (1 $\mu$g/ml) was added to half the cultures. Control experiments were set up with bone cultures alone in medium, with and without indomethacin. After 3 days' culture, the pH of all the culture media was measured and the tumour fragments were processed routinely for histology.

*Squamous-carcinoma cell lines.*—Six tumour cell lines derived from squamous carcinomas of the head and neck were examined. Details of the establishment and properties of these and other squamous-

TABLE I.—*Squamous-carcinoma cell lines, LICR(Lond.)/HN1–6*

| LICR/HN cell line* | Origin† | Months in culture |
|---|---|---|
| 1 | M.51 Tongue | 33 |
| 2 | M.49 Larynx | 28 |
| 3 | M.63 Tongue | 27 |
| 4 | M.57 Larynx | 29 |
| 5 | M.73 Tongue | 12 |
| 6 | M.54 Tongue | 12 |

* Nature of the cell lines confirmed by karyotype analyses, ultrastructural appearances, and capacity to grow as xenografts in immune-deprived CBA mice.

† M = male. Numbers represent age of patient. Specimens obtained at the time of radical surgery (glossectomy and radical neck dissection, total laryngectomy) for recurrent or radiation-resistant primary tumours.

carcinoma lines will be reported separately (Easty *et al.*, 1981) but their salient features are shown in Table I.

The establishment of the carcinoma cell lines may be briefly summarized as follows. Fresh tumours were collected in theo perating theatre and reduced to 2mm$^3$ fragments. Five to 6 fragments from each tumour were then incubated for 5–7 days without disturbance in 2·5ml Dulbecco's modified Eagle's medium with 10% foetal calf serum (FCS) in 25cm$^2$ flasks to permit attachment of explants. Fibroblasts were eliminated either by mechanical scraping or with a complement-dependent, cytotoxic monoclonal antifibroblast antibody (LICR LON/FIB 8b; Edwards *et al.*, 1980). Any residual normal epithelium was lost through senescence and, in successful cultures, a pure growth of proliferating squamous-carcinoma cells remained which could be serially subcultured.

For *in vitro* osteolysis assays, tumour cells from the 6 lines were grown to confluence in 174cm$^2$ culture flasks in Dulbecco's modified Eagle's medium supplemented with 10% FCS (Gibco, Europe) and antibiotics (100 μg/ml benzyl penicillin, 2·5 μg/ml minocycline) at 37°C in 10% $CO_2$ in air. They were then washed ×3 with Bigger's medium and incubated with fresh Bigger's medium supplemented with 5% heat-inactivated rabbit serum for 24 h. Cell-free supernatants, prepared by Millipore filtration (0·45 μm), were collected, pH-adjusted and assayed for osteolytic activity. The remaining cell layers were trypsinized and cell densities determined in a haemacytometer cell. The pH of the test and control media was measured at the end of each 3-day assay.

The effects of indomethacin were studied by including the drug (1 μg/ml) in the medium for the initial 24h period of incubation of cell cultures in modified Bigger's medium.

Three fibroblastoid cell lines derived from primary cultures of squamous carcinomas were also assayed for bone-resorbing activity.

Calvaria were examined histologically after 3 days' incubation in test and control cultures. The specimens proved to be too fragile for routine histological processing, but satisfactory results were obtained with material fixed in formol–saline, decalcified in EDTA, embedded in methacrylate resin, cut at 1–2μm and stained with toluidine blue, haematoxylin and eosin (HE) and methyl green–pyronine (MGP).

### RESULTS

### In vitro *osteolysis by fresh squamous carcinoma*

The *in vitro* osteolytic activity of 8 primary squamous carcinomas is summarized in Table II and Fig. 1. Four of the

TABLE II.—In vitro *osteolysis by fresh squamous carcinomas*

| Patient | Site of primary tumour | Amount of tumour (wet st. mg) | Indo-methacin | $^{45}$Ca release (test/control ratios) | ΔpH | P* |
|---|---|---|---|---|---|---|
| Il. | Pyriform fossa | 113 | − | 2·49 ± 0·23 | 0·08 | < 0·01 |
| M.62 | | 113 | + | 1·91 ± 0·13 | 0·08 | < 0·05 |
| Conc. | Oropharynx | | − | 2·01 ± 0·06 | 0·02 | < 0·01 |
| F.54 | | | + | 1·17 ± 0·11 | 0·05 | < 0·01 |
| Ahm. | Tongue | | − | 1·81 ± 0·08 | 0·17 | < 0·01 |
| F.58 | | | + | 1·30 ± 0·10 | 0·24 | < 0·01 |
| Tsim. | Floor of mouth | 75 | − | 1·69 ± 0·22 | 0·11 | < 0·05 |
| M.65 | | 74 | + | 1·48 ± 0·15 | 0·10 | N.S. |
| Pat. | Hypopharynx | | − | 1·30 ± 0·13 | −0·01 | < 0·02 |
| M.57 | | | + | 1·03 ± 0·05 | 0·01 | N.S. |
| Hal. | Pyriform fossa | 38 | − | 1·19 ± 0·09 | −0·03 | N.S. |
| M.63 | | 42 | + | 1·03 ± 0·03 | −0·09 | N.S. |
| Led. | Nasal septum | 53 | − | 1·16 ± 0·09 | 0·06 | N.S. |
| M.64 | | 46 | + | 1·05 ± 0·07 | 0·00 | N.S. |
| Delv. | Oropharynx | 74 | − | 1·12 ± 0·03 | 0·03 | < 0·01 |
| F.44 | | 73 | + | 1·10 ± 0·04 | 0·05 | N.S. |

* Calculated according to Student's *t* test: significant level 0·05.
N.S. = not significant.

FIG. 1.—*In vitro* bone-resorbing activities of 8 freshly excised squamous carcinomas of the head and neck with (stippled) or without (blank) 1 μg/ml indomethacin. There are 5 carcinomas of the oropharynx (Il, Conc., Pat., Hal., Delv.) and single examples of carcinomas of the tongue (Ahm.), floor of mouth (Tsim.) and nasal septum (Led.). Each column represents the mean ± s.e. osteolytic activity of 4 pairs of test and control cultures.

tumours showed moderate to marked bone resorption, measured by the release of $^{45}Ca$ from isotopically labelled calvaria. Osteolytic activity was less in all the cultures to which the PG-synthesis inhibitor, indomethacin, was added; in 5 of the tumours tested, this indomethacin-associated decrease in bone resorption was greater than 50%. Variations in pH of the culture media in the course of the experiments were small (see Table II).

The cultured tumour fragments were well preserved and their histological appearances were comparable to those of the tumour in the main surgical specimens, consisting of variable amounts of squamous-carcinoma cells, fibrous stroma, mixed inflammatory cells, granulation tissue and necrotic debris (Fig. 2).

### In vitro *osteolysis by squamous-carcinoma cell lines*

Six established squamous-carcinoma cell lines (LICR(Lond.)/HN1–6) were examined. HN2 and 4 were derived from 2

TABLE III.—In vitro *osteolysis by squamous-carcinoma cell lines*

| LICR/HN cell line | Cell density (× $10^5$/ ml) | $^{45}Ca$ release (test/control) | ΔpH | *P* |
|---|---|---|---|---|
| 4 M.57 Larynx | 1·9 | 2·21 ± 0·10 | −0·02 | < 0·001 |
| 6 M.54 Tongue | 2·9<br>3·4<br>2·2 | 1·87 ± 0·14<br>2·02 ± 0·22<br>1·76 ± 0·09 | 0·09<br>0·07<br>0·14 | < 0·01<br>< 0·01<br>< 0·01 |
| 2 M.49 Larynx | 9·5<br>11·5 | 1·50 ± 0·08<br>1·55 ± 0·09 | −0·02<br>0·02 | < 0·01<br>< 0·01 |
| 5 M.49 Tongue | 2·6<br>3·3<br>3·2 | 1·28 ± 0·08<br>1·59 ± 0·07<br>1·54 ± 0·13 | 0·05<br>0·15<br>0·11 | < 0·02<br>< 0·01<br>< 0·02 |
| 1 M.51 Tongue | 1·9<br>1·9 | 1·35 ± 0·14<br>1·52 ± 0·13 | −0·3<br>0·07 | N.S.<br>< 0·01 |
| 3 M.63 Tongue | 4·2<br>3·5 | 1·41 ± 0·07<br>1·51 ± 0·07 | 0·12<br>0·13 | < 0·01<br>< 0·01 |
| FB 1 | | 0·85 ± 0·05 | −0·05 | N.S. |
| FB 2 | 2·7 | 1·07 ± 0·09 | 0·06 | N.S. |
| FB 3 | 2·2 | 1·61 ± 0·14 | 0·00 | < 0·02 |

FIG. 2.—A,B. Fragments of two squamous carcinomas of the oropharynx after 3 days' culture. The tumour cells are well preserved. Note the admixture of neoplastic and host stromal elements. Both H. & E. × 200.

squamous carcinomas of the larynx, and the remaining 4 lines from squamous carcinomas of the tongue (see Table I). The results of *in vitro* osteolysis assays, using the supernatant culture media from these 6 cell lines, are presented in Table III and Fig. 3.

Supernatants from 2 of the cell lines—HN4 and 6—produced marked bone resorption. Culture media from the remaining 4 cell lines were moderately active, as were the media from one of the 3 control fibroblastoid cell lines (Fig. 3). The results of experiments with each cell line were reproducible on 2 or 3 separate occasions. Variations in pH of the culture medium in the course of the assays were small (see Table III).

Morphological changes were examined in 4 pairs of test and control calvaria. The slides were coded beforehand, but histological differences between the 2 groups were readily apparent (Fig. 4). Bones incubated for 3 days in control media were smooth and the trabeculae appeared intact. Multinucleate osteoclasts were infrequently seen. After incubation for 3 days with media from the carcinoma-cell lines, the calvaria were soft and there was obvious loss of bone substance. Multi-

FIG. 3.—*In vitro* bone-resorbing activities of supernatant media from 6 established squamous-carcinoma cell lines: HN1, 3, 5 and 6—carcinomas of the tongue; HN2, 4—carcinomas of the larynx. Media from 3 fibroblastoid cell lines (FB) are also included. Each column represents the mean ± s.e. osteolytic activities of 4 pairs of test and control cultures.

nucleate osteoclasts were conspicuous. Several mononuclear cells which were not osteoblasts were observed lying close to the internal bone surfaces.

The effects of previous culture with *indomethacin* on subsequent bone resorption were investigated in media from 4 squamous-carcinoma cell lines—HN2, 3, 4 and 5. The results, shown in Table IV and Fig. 5, differ from those described earlier in relation to indomethacin and fresh squamous carcinomas.

TABLE IV.—In vitro *osteolysis by* 4 *squamous-carcinoma cell lines: effects of indomethacin* (1 *μg/ml*)

| LICR/HN cell line | Cell density ($\times 10^5$/ml) | Indo-methacin | $^{45}$Ca release (test/control) | ΔpH |
|---|---|---|---|---|
| HN4 | 1·9 | − | 2·21 ± 0·10 | −0·02 |
| | 2·3 | + | 2·18 ± 0·10 | 0·15 |
| | 2·0 | + | 2·03 ± 0·14 | 0·05 |
| HN2 | 11·5 | − | 1·55 ± 0·09 | 0·02 |
| | 9·4 | + | 1·30 ± 0·04 | 0·03 |
| HN5 | 3·2 | − | 1·54 ± 0·13 | 0·11 |
| | 3·4 | + | 1·89 ± 0·05 | 0·13 |
| HN3 | 3·5 | − | 1·51 ± 0·07 | 0·13 |
| | 3·7 | + | 1·54 ± 0·05 | 0·10 |

No differences in $^{45}$Ca release statistically significant.

The effects of indomethacin varied in different cell lines: *in vitro* bone resorption was less in Lines HN4 and 2, unchanged in HN3 and apparently greater in HN5. The most obvious explanation for the failure of indomethacin to inhibit bone resorption in this and in "indomethacin-resistant" cultures is that the osteoclasts are stimulated by factors other than PGs (see Discussion).

### DISCUSSION

Previous histological examination of surgical specimens suggests that osteoclasts play an important role in the spread of squamous carcinomas of the head and neck into local bone (Carter *et al.*, 1980; Carter & Pittam, 1980; Carter & Tanner, 1979). These morphological features are not specific, and the changes observed are comparable to those associated with the growth of blood-borne skeletal metastases of various kinds (Galasko, 1975, 1976). Similar findings have also been reported in experimental tumours implanted in or near bone (Faccini, 1974) though there is controversy as to whether the morphological changes observed are confined to

FIG. 4.—Fragments of neonatal mouse calvaria after 3 days' culture. A. Control calvarium. The bone trabeculae are well preserved with no multinucleate osteoclasts. B. Test calvarium. The bone substance is lost and multinucleate osteoclasts are prominent. Both H. & E. ×750.

the vicinity of the tumour (Galasko, 1976; Galasko & Bennett, 1976; Young *et al.*, 1975; Hough *et al.*, 1977; Wolfe *et al.*, 1978).

The present paper is concerned with some of the mechanisms whereby osteoclasts in the vicinity of squamous carcinomas may be activated. A standard *in vitro* bone-resorption assay has been used (Reynolds, 1968) which provides reproducible and accurate results. The short-term organ cultures and the tumour-cell lines provide complementary approaches to the analysis of tumour-associated osteolysis, but the two systems differ in certain respects. The fragments of fresh tumour presumably provide a continuous source of osteolytic factors for most or all of the 3 days that they are maintained in culture. By contrast, bone-resorbing activity released into the supernatant medium by established tumour-cell lines presumably ceases after the cells have been removed by millipore filtration and

FIG. 5.—*In vitro* bone-resorbing activities of supernatant media from 4 established squamous-carcinoma cell lines, co-cultivated for 24 h with 1 mg/ml indomethacin (stippled) or without (blank). Each column represents the mean ±s.e. osteolytic activity of 4 pairs of test and control cultures.

the assays set up with the cell-free supernatants. The effects of indomethacin also require careful interpretation. The drug inhibits PG synthesis but it is not strictly possible in our test systems to define the source of any PGs produced. They can be derived from the tumour cells or from the bone itself in response to a variety of agents as diverse as antibodies, proteolytic enzymes and growth factors (Raisz *et al.*, 1974; Dowsett *et al.*, 1976; Tashjian & Levine, 1978).

Short-term (3-day) cultures of fresh squamous carcinomas evoked a variable degree of osteolysis; activity was marked in 4 of the 8 tumours examined and, in all of them, bone resorption was clearly inhibited by indomethacin. These observations indicate that PGs are likely to be involved, and the findings are compatible with results from fresh squamous carcinomas of the head and neck (Bennett *et al.*, 1980). The evidence that PGs are released *in vitro* and induce bone resorption is, however, still indirect, and work is in progress to separate and characterize these indomethacin-sensitive factors. It is also essential that bone resorption is examined in more control tissues. The choice of such material is difficult, and at present we are collecting tissues from uninvolved resection lines from major surgical specimens and from ostensibly normal tissues removed in the course of dental and plastic procedures. It is stressed that the tumour fragments consist of a mixture of neoplastic and stromal elements, and that PGs and other "tumour-associated products" may be derived from several different cell types. Host cells, in this context, may also include bone cells themselves which have been shown to elaborate PGs—*vide supra.*

These non-homogeneous fragments of fresh tumour correspond reasonably closely to the carcinomas in the patients from which they were obtained; but for a closer analysis of bone destruction it is necessary to examine the osteolytic capacity of carcinoma cells alone. These experiments have now been done with a series of squamous-carcinoma cell lines "uncontaminated" by host elements. Ten tumour cell lines have been established by us (Easty *et al.*, 1981) and supernatant culture media from 6 lines have been assayed for osteolytic activity. A variable degree of *in vitro* bone resorption was demonstrated. The effects of indomethacin in this system were complex. Instead of a uniform inhibition of osteolysis, culture with indomethacin produced a variety of effects, with bone resorption decreased, unchanged or possibly, in one line, enhanced. Previous work (Levine *et al.*, 1972; Dowsett *et al.*, 1976) has shown that indomethacin, tested at the same concentration and for the same length of time as in our own experiments, reduced the *in vitro* production of PGs from high levels to control values. More squamous-carcinoma cell lines need to be examined, but it is stressed that all lines with osteolytic activity evoked an osteoclastic response in the calvaria which were exposed to their supernatant culture media. It therefore appears that most of the lines so far studied release indomethacin-*resistant* (non-PG) factors. Various osteoclast-activating factors other than PGs have been described but their identity and mode of action remain unclear (Horton *et al.*, 1972; Mundy *et al.*, 1974; Luben, 1980). Some authors regard "osteoclast-activating factor" as a lymphokine, the secretion of which may be modified by PGs (Gordon *et al.*, 1976). Isolation and chemical characterization of the indomethacin-sensitive PGs associated with the carcinoma cell lines is in progress.

The local accumulation of osteoclasts observed on the eroding bone surface in surgical specimens is simulated in the test calvaria maintained *in vitro*. Similar accumulation in an *in vitro* system has recently been reported by Schelling *et al.* (1980). It is not clear whether osteoclasts in these circumstances are redistributed or increased in number (see Schelling *et al.*, 1980); accurate estimates of all the osteolysing cells are, in our view, difficult to make. Multinucleate osteoclasts can measure up to 80 $\mu$m in diameter, and different parts of the same cell may appear, discontinuously and cut obliquely, in different parts of thin (1–2$\mu$m) sections. In addition, mononuclear cells can probably also destroy bone (Mundy *et al.*, 1977; Kahn *et al.*, 1978; McArthur *et al.*, 1980). Mononuclear cells were regularly seen by us on the endosteal surfaces of resorbing calvaria, but their identity, origin, and function are obscure.

The work described here has concentrated solely on certain osteoclast-mediated components of local bone destruction associated with squamous carcinomas of the head and neck. Lysosomal hydrolases and other proteinases, tumour- or host-cell-derived, which may be concerned with the direct breakdown of the organic bone matrix, have not been considered (Dowsett *et al.*, 1976; Eilon & Mundy, 1978; Heersche, 1978). Two conclusions can be drawn at this stage:

(1) Stimulants for osteoclastic activity are derived from both carcinoma cells and from host cells in the tumour stroma.

(2) These stimulants are diverse. Indomethacin-*sensitive* agents, presumed to be PGs, are most convincingly demonstrated in the fresh tumours. Indomethacin-*resistant* agents, presumed to be substances other than PGs, are more characteristic of the homogeneous carcinoma cell lines.

The clinical implications of PGs and other products associated with squamous carcinomas of the head and neck are being followed. The short (3-day) cultures, in particular, are comparatively simple to handle and they provide results within a week of surgery. It is improbable that PGs and related substances will be useful in specifically predicting bone invasion as this can usually be assessed clinically, but some other prognostic significance for these products may become apparent (*cf.* Fitzpatrick & Stringfellow, 1979; Rolland *et al.*, 1980).

We are indebted to Dr V. M. Dalley, Mr P. Clifford, FRCS, and Mr H. J. Shaw, FRCS, for access to clinical material; to Mr N. S. B. Tanner, FRCS, and Mr M. R. Pittam, FRCS, for collecting fresh tumour tissue; to Mrs Diana Mitchell, Mr J. D. B. Roberts and the staff of the histology laboratory; and to Mr K. G. Moreman for preparing the illustrations. S.W.T. is in receipt of a Postgraduate Scholarship awarded by the Shell Trading Company. R.L.C. acknowledges financial support from the Medical Research Council.

### REFERENCES

Bennett, A., Carter, R. L., Stamford, I. F. & Tanner, N. S. B. (1980) Prostaglandin-like material extracted from squamous carcinomas of the head and neck. *Br. J. Cancer*, **41**, 204.

Carter, R. L. & Pittam, M. R. (1980) Squamous carcinomas of the head and neck: Some patterns of spread. *J. R. Soc. Med.*, **73**, 420.

Carter, R. L. & Tanner, N. S. B. (1979) Local invasion by laryngeal carcinoma—the importance of focal (metaplastic) ossification within laryngeal cartilage. *Clin. Otolaryngol.*, **4**, 283.

Carter, R. L., Tanner, N. S. B., Clifford, P. & Shaw, H. J. (1980) Direct bone invasion in squamous carcinomas of the head and neck: Pathological and clinical implications. *Clin. Otolaryngol.*, **5**, 107.

Dowsett, M., Eastman, A. R., Easty, D. M., Easty, G. C., Powles, T. J., & Neville, A. M. (1976) Prostaglandin mediation of collagenase-induced bone resorption. *Nature*, **263**, 72.

Easty, D. M., Easty, G. C., Carter, R. L., Monaghan, P. & Butler, L. J. (1981) Ten human carcinoma cell lines derived from squamous carcinoma of the head and neck. *Br. J. Cancer*. (In press.)

Edwards, P. A. W., Easty, D. M. & Foster, C. S. (1980) Selective culture of epithelioid cells from a human squamous carcinoma using a monoclonal antibody to kill fibroblasts. *Cell Biol. Int. Rep.*, **4**, 917.

EILON, G. & MUNDY, G. R. (1978) Direct resorption of bone by human breast cancer cells *in vitro*. *Nature*, **276**, 726.

FACCINI, J. M. (1974) The mode of growth of experimental metastases in rabbit femora. *Virchows Arch. A.* [*Pathol. Anat.*], **364**, 249.

FITZPATRICK, F. A. & STRINGFELLOW, D. A. (1979) Prostaglandin $D_2$ formation by malignant melanoma cells correlates inversely with cellular metastatic potential. *Proc. Natl Acad. Sci. U.S.A.*, **76**, 1765.

GALASKO, C. S. B. (1975) The pathological basis for skeletal scintigraphy. *J. Bone Jt Surg.*, **57B**, 353.

GALASKO, C. S. B. (1976) Mechanisms of bone destruction in the development of skeletal metastases. *Nature*, **263**, 507.

GALASKO, C. S. B. & BENNETT, A. (1976) Relationship of bone destruction in skeletal metastases to osteoclast activation and prostaglandins. *Nature*, **263**, 508.

GORDON, D., BRAY, M. A. & MORLEY, J. (1976) Control of lymphokine secretion by prostaglandins. *Nature*, **262**, 401.

HEERSCHE, J. N. M. (1978) Mechanism of osteoclastic bone resorption: a new hypothesis. *Calcif. Tissue Res.*, **26**, 81.

HORTON, J. E., RAISZ, L. G., SIMMONS, H. A., OPPENHEIM, J. J. & MERGENHAGEN, S. E. (1972) Bone resorbing activity in supernatant fluid from cultured human peripheral blood leukocytes. *Science*, **177**, 793.

HOUGH, A., JR, SEYBERTH, H., OATES, J. & HARTMANN, W. (1977) Changes in bone and bone marrow of rabbits bearing the VX2 carcinoma. *Am. J. Pathol.*, **87**, 537.

KAHN, A. J., STEWART, C. C. & TEITELBAUM, S. L. (1978) Contact-mediated bone resorption by human monocytes *in vitro*. *Science*, **199**, 988.

LEVINE, L., HINKLE, P. M., VOELKEL, E. F. & TASHJIAN, A. H., JR (1972) Prostaglandin production by mouse fibrosarcoma cells in culture: Inhibition by indomethacin and aspirin. *Biochem. Biophys. Res. Comm.*, **47**, 888.

LUBEN, R. A. (1980) An assay for osteoclast-activating factor (OAF) in biological fluids. *Cell. Immunol.*, **49**, 74.

MCARTHUR, W., YAARI, A. M. & SHAPIRO, I. M. (1980) Bone solubilization by mononuclear cells. *Lab. Invest.*, **42**, 450.

MUNDY, G. R., LUBEN, R. A., RAISZ, L. G., OPPENHEIM, J. J. & BUELL, D. N. (1974) Bone-resorbing activity in supernatants from lymphoid cell lines. *N. Engl. J. Med.*, **290**, 867.

MUNDY, G. R., ALTMAN, A. J., GONDEK, M. D. & BANDELIN, J. G. (1977) Direct resorption of bone by human monocytes. *Science*, **196**, 1109.

RAISZ, L. G., SANDBERG, A. L., GOODSON, J. M., SIMMONS, H. A. & MERGENHAGEN, S. E. (1974) Complement-dependent stimulation of prostaglandin synthesis and bone resorption. *Science*, **185**, 789.

REYNOLDS, J. J. (1968) Inhibition by calcitonin of bone resorption induced *in vitro* by Vitamin A. *Proc. R. Soc. B*, **170**, 61.

ROLLAND, P. H., MARTIN, P. M., JACQUEMIER, J., ROLLAND, A. M. & TOGA, M. (1980) Prostaglandin in human breast cancer: Evidence suggesting that an elevated prostaglandin production is a marker of high metastatic potential for neoplastic cells. *J. Natl Cancer Inst.*, **64**, 1061.

SCHELLING, S. H., WOLFE, H. J. & TASHJIAN, A. H., JR (1980) Role of the osteoclast in prostaglandin-$E_2$-stimulated bone resorption. *Lab. Invest.*, **42**, 290.

TASHJIAN, A. H., JR, & LEVINE, L. (1978) Epidermal growth factor stimulates prostaglandin production and bone resorption in cultured mouse calvaria. *Biochem. Biophys. Res. Comm.*, **85**, 996.

WOLFE, H. J., BITMAN, W. R., VOELKEL, E. F., GRIFFITHS, H. J. & TASHJIAN, A. H., JR (1978) Systemic effects of the VX2 carcinoma on the osseous skeleton. *Lab. Invest.*, **38**, 208.

YOUNG, D. M., FIORAVANTI, J. L., PRIEUR, D. J. & WARD, J. M. (1975) Hypercalcaemic VX2 in rabbits: A clinicopathologic study. *Lab. Invest.*, **35**, 30.